# Innovating for Sustainability: Attributes, Motivations, and Responsibilities in the Finnish Food Packaging Ecosystem

**DOI:** 10.1007/s43615-022-00217-2

**Published:** 2022-10-18

**Authors:** Lotta Ruippo, Hanna Koivula, Jaana Korhonen, Anne Toppinen, Eliisa Kylkilahti

**Affiliations:** 1 Faculty of Agriculture and Forestry, Department of Economics and Management, University of Helsinki, P.O. 27, 00014 Helsinki, Finland; 2 Faculty of Agriculture and Forestry, Department of Food and Nutrition, University of Helsinki, Helsinki, Finland; 3 Oak Ridge Institute for Science and Education (ORISE), Oak Ridge, USA; 4 Faculty of Agriculture and Forestry, Department of Forest Sciences, University of Helsinki, Helsinki, Finland; 5 Helsinki Institute of Sustainability Science (HELSUS), University of Helsinki, Helsinki, Finland

**Keywords:** Packaging, Sustainability, Innovation ecosystem, Food

## Abstract

Without food packaging, the global food supply chain could not function. Packaged food products are protected in transportation and retail, reducing food waste. Packaging is also a visible feature of environmental debates, as concerns over solid waste have become a part of public discussion. Responding to both challenges requires the packaging sector to develop and adopt sustainable innovations. This study seeks to understand the role of sustainability in food packaging innovation through expert interviews. The results of this study aid in clarifying actor roles for innovation processes in the packaging sector through encouraging collaboration and integrating socioeconomic dimensions of sustainability into innovation. The findings suggest that actors are primarily concerned with the ecological sustainability of packaging while economic or social factors in sustainable innovation play a more minor role. Finally, the study finds that much responsibility over developing innovations is placed on governmental organizations and brand owners in the food and beverage industry.

## Introduction

The global food packaging market is valued at around 303 billion US dollars, with forecasted growth until 2027 [1]. The scale of packaging production is vast—according to Korhonen et al. [2], 3.4 trillion units of packaging were produced in 2016 alone. It is estimated that 57 percent of packaging is used for consumer products, of which food and beverage packaging represents about 38 percent [3]. The linear, single-use economy that still largely exists around food packaging does have its purpose, as the primary duty of packaging is to contain, protect, and inform [4]. Furthermore, the largest associated environmental impacts of packaging have to do with food waste, rather than with municipal solid waste. For example, a study by Yokokawa et al. [5] suggests that additional use of packaging materials may even be justified, if food waste is reduced through, e.g., a reduction in portion sizes or an increase in the self-life of a product. In addition, excessive packaging sizes [6] and loss of protective capabilities [7] have both been identified as culprits in increasing the creation of food waste. It is important to emphasize that food losses and waste occur throughout the value chain, but waste at the household level incorporates the cumulative environmental impacts of food production [8]. Devoting more attention to the packaging value chain is crucial, as its environmental impact is multifaceted and very dependent on the product-packaging dynamic.

Public anxieties regarding plastic pollution are often linked to the use of food packaging, which remains the most common application of plastic. Ninety-five percent of plastic packaging is discarded after first use [9]. What is more, plastic use has social implications—as Barford and Ahmad [10] discuss, the circular economy of plastics in low- and middle-income countries is very dependent on waste pickers who have very weak social protections, low wages, and dangerous working conditions. It has been estimated that approximately 79 percent of all plastic that has ever been produced is either in the landfill or in nature [11], highlighting the importance of efforts to implement circular economy innovations into the packaging sector in a socially sustainable manner.

The needs that food packaging serves are changing rapidly. The growth of online grocery shopping, demand for convenient and pre-packaged food, ageing population, and decreasing household sizes all affect the types of packaging that are needed [12]. A growing demand for take-away dining has also been identified in literature [13]. At the same time, the supply of non-renewable materials has become more volatile, and new technologies for packaging design and production have been introduced [14]. Packaging alternatives are in development and entering the market: according to Han et al. [15], these are often related to raw material innovation, production process innovation, and end-of-life innovation. Other characteristics of sustainable packaging include sustainability of materials, ensuring functionality, food safety, and mitigation of food loss and waste [16].

Adopting new packaging innovations is difficult: as argued by Werner et al. [17], technology transfer across institutions in the packaging system is slow, technologies are difficult to scale up, and the regulation of packaging—particularly food safety related ones—is stringent. Moreover, the success of a food packaging innovation is ultimately dependent on consumer acceptance, amidst increasingly negative perceptions about packaging use. Understanding and bringing about circular economy transitions in packaging production also likely requires stakeholder collaboration [18]. Although food packaging has received a lot of attention in literature, the focus is commonly on the consumer of food packaging, or the packaging itself in the form of life cycle analysis, material technology, or waste management. Literature on sustainable innovation is often process-oriented. Thus, asking if the innovations lead to improved sustainability receives less attention [19]. Therefore, exploring the perceptions of actors who impact product development of companies in the sector has been less common. Integrating these perspectives is important, as the mindsets and ideas of such actors are inexorably enabling or prohibiting sustainable innovations in the market.

Finland provides an interesting case study into the sustainable packaging industry due to the economic importance of its forest industry, which represented 18 percent of the value of exports in 2020 [20]. Therefore, there are significant interests in developing innovative, wood or fiber-based packaging alternatives for the food and beverage sector that simultaneously needs to comply with food safety standards and increasing sustainability requirements. This paper examines how experts in the packaging ecosystem make sense of these complexities, as this could impact the direction of the industry.

This paper explore sustainable packaging innovations from the perspective of actors in the packaging ecosystem. In order to complement existing literature at the intersection of new technologies, consumer behavior, and packaging design, the study examines notions held by experts involved in the packaging sector. The study discusses the elements of sustainable packaging named by the actors themselves, the motivations behind innovative packaging, and the distribution of responsibility in the packaging innovation ecosystem. The study aims to answer the following questions:

1. What attributes of packaging are considered sustainable among actors operating in the core of the packaging innovation ecosystem?
2. What motivates these actors to create sustainable packaging innovations?
3. How is responsibility shared among actors in the innovation ecosystem?

By examining these three questions, it is possible to understand the prioritizations of sustainability in packaging and the reasonings driving innovation within the ecosystem.

## Literature Review

Innovations do not occur in a vacuum; they demand a range of skills, knowledge, and resources [21]. Bringing these together often requires in-depth collaboration, which is why literature on innovation ecosystems has sought to elucidate the role of collaborative actors and institutions. If done correctly, the coming together of skills, knowledge, and resources from a variety of actors could bring about the problem-solving innovations that the planet is in dire need of [22]. Similarly, others argue that accelerating sustainable innovation requires more in terms of stakeholder collaboration, as the goals of sustainable innovation are three-fold [23, 24]. Sustainable innovations are typically defined as innovations that incorporate the ecological, economic, and social attributes of innovation [25, 26]. Recent studies emphasize that sustainable innovations should improve products on a fundamental or systemic level, rather than aiming for optimization of single elements. However, due to the three-fold nature of sustainable innovation, they tend to be highly complex in their effects [23].

Sustainable innovations are typically defined as innovations that incorporate the ecological, economic, and social dimensions of innovation [25, 26]. Conceptualizations of sustainable innovation can emphasize results beyond economic value—for example, Oksanen and Hautamäki [22] argue that economic benefits have a mere instrumental value, and sustainable well-being and sustainable development are much more important. Moreover, the authors emphasize that sustainable innovations improve products on a fundamental or systemic level, rather than aiming to optimize singular facets. However, due to the three-fold nature of sustainable innovation, they tend to be highly complex in their effects [23].

An innovation ecosystem is an evolving interconnected system of actors, their activities, relations, and outputs (Grandstrand and Holgersson 2020). Here, the term innovation ecosystem is simply understood as the arrangements, through which firms work to offer solutions to their customers (adapted from [21]). As a concept, innovation ecosystem tends to focus on value creation whereas the more traditional concept of business ecosystem places emphasis on value capture [27]. The concept of an innovation ecosystem is not often applied to the packaging industry in academic literature, but here, it is used to emphasize the complexity in the network of actors and their interactions in creating value through innovations in materials, services, and so forth.

The packaging industry is not a single line of business, but a network of actors in multiple stages of the value chain from raw material production to waste management. The literature reflects this complexity, as studies have examined consumer perceptions [28, 29], the life-cycle impacts of food packaging (e.g. [7]; [30–32]) and the governance of the packaging industry [33, 34]. Studies into the governance of food packaging have concluded that the governance of food packaging is challenging because of surrounding ambiguity; food packaging governance exists in-between food, plastics, and circular economy discussions [33]. The environmental impacts under focus also vary from marine plastic pollution to extraction of raw materials, health, and climate change [34]. In order to fully capture the complexity of the packaging ecosystem, perspectives from actors beyond the corporate world are needed.

The packaging innovation ecosystem also affects adoption of novel products. Tiekstra et al. [16] argue that the challenges in the adoption of active packaging is not specific to any one actor—rather, they run across the entire value chain, and affect a range of stakeholders in packaging innovation. Therefore, the sector could potentially gain from more in-depth collaboration. For example, Keränen et al. [23] examine the diffusion of sustainable innovation in food packaging value networks, with a focus on bioplastics. They identify that the changes to the existing value network and their connections behind the successful diffusion of sustainable innovation take place at three levels—firm, network, and macro.

Creation and diffusion of innovations into the markets requires public and private investment in partnerships and strategic foresight on the most desirable attributes of packaging in the future [35, 36]. Yet, scaling up sustainable packing innovations in the global markets requires that the supply networks are reliable, and the risks of disruptions are reduced by creating geographically dispersed ecosystems. Viitanen et al. [33] discuss how large-scale food and drink retailers do not take the risk of being dependent on the supply from a single factory.

Consumers are not an insignificant force in food packaging development: for example, Vernuccio et al. [37] list consumer behavior as the first driver of food packaging innovation, along with new environmental values, and technological development. Regardless of the consumer interest in environmental sustainability, other attributes that have a role in consumer decision-making include the price and quality of a product. Russell [38] argues that food packaging has an important role in supporting the sustainability of the overall food system, but this is challenged by negative consumer perceptions of food packaging. Furthermore, consumers do not often possess the ability to judge the sustainability of packaging, and can be misled by, for example, packaging design [28]. Lindh et al. [29] also found that consumers tend to seldom think the sustainability of packaging materials beyond attributes related to environmental sustainability, which in their study, usually meant consumers preferred paper-based packaging materials. Russell [24] argues that consumers tend to interact with packaging when purchasing food or discarding packaging waste—this only offers a narrow look into the consumer-packaging interaction. Literature on consumer perceptions of packaging should be supported with studies into the value chains of the packaging industry.

This study is particularly interested in examining the three related questions regarding sustainability and how they appear in food packaging innovation. Examining their role in food packaging innovation can be useful, as implementing sustainability into innovation processes may aid in solving global challenges [30]. As discussed above, food packaging interlinks several complex issues from marine plastic pollution to food loss and waste in the food supply chain, offering an interesting look into the persistent sustainability issues innovation could seek solutions to. Innovations do not, however, occur in a vacuum. They demand a range of skills, knowledge, and resources [21]. Bringing these together often requires in-depth collaboration, which is why literature on innovation ecosystems has sought to elucidate the role of collaborative actors and institutions. If done correctly, the coming together of skills, knowledge, and resources from a variety of actors could bring about the problem-solving innovations that the planet is in dire need of [22].

## Materials and Methods

This study adopts a qualitative approach to study sustainability in food packaging innovation. Due to the very context-specific nature of the Finnish food packaging ecosystem and its stakeholders, qualitative research was deemed appropriate [39]. Conducting such a case study is useful, as it allows for a range of sources. Case studies also allow for certain context awareness, as the results are likely affected by the unique research setting [40].

Fourteen semi-structured expert interviews were conducted for the study in 2020. While some time has passed between data collection and the publication of this article, the knowledge gathered is still very relevant. Here, the goal is to describe and analyze the overarching perceptions that guide packaging value chains rather than opinions on current legislation. The participants were individuals who were involved in food packaging innovation either through their role in the private sector or the public sector—in this case, governance or research. Actors outside business were also included, as the study interviewed experts from the government and research. The participants in the empirical analysis include public servants, directors of industry associations, and representatives of various enterprises within the Finnish food packaging innovation ecosystem. That is, the interviewees were selected based on their knowledge of the subject matter of the study. Purposeful sampling was used to select information-rich participants, which often is the reasoning for this method of sampling [41]. All participants held senior positions within their respective organizations (see Table 1). Interviews are very useful, when participants can influence the outcomes of processes around them [42]. Moreover, choosing semi-structured interviews over structured ones allowed for clarifications and follow-up questions in this case [43]. This provided a reason to seek out informants particularly from managerial positions.

**Table 1:** Description of interviewees, identification numbers, and the interview setting

ID	Position	Remote	Length
1	CEO, Material production	Yes	36 min
2	CEO, Industry association	Yes	50 min
3	Senior specialist, Finnish government	Yes	33 min
4	Chief business development officer, Material production	Yes	57 min
5	Senior advisor, Finnish government	Yes	40 min
6	Head of packaging development, Brand owner	Yes	39 min
7	CEO, Waste management	Yes	27 min
8	Head of packaging development, Brand owner	No	41 min
9	CEO, Packaging producer	No	39 min
10	CEO, Material production	Yes	46 min
11	Scientist, Research organization	No	67 min
12	CEO, Packaging producer	Yes	27 min
13	Head of sales, Brand owner	Yes	32 min
14	CEO, Industry association	No	60 min

Private sector participants included brand owners in the food and beverage industries, material producers, and packaging producers. The enterprises vary from small and medium sized to multinational corporations. Actors from waste management and relevant industry associations were also included in the sample. All actors represent different organizations. The interviewees from the government work in separate branches. Specific titles have been left out to protect the anonymity of the interviewees. The interviews were discontinued when saturation was reached, and no new important information seemed to be emerging.

All interviews were conducted in spring 2020. Most of the interviews had to be moved to a remote setting due to the SARS-CoV 2 pandemic and work-from-home orders as can be observed from Table 1. All participants were sent the interview guide (Appendix) upon meeting request, and consent was requested for the recording. The interviews were recorded and transcribed. They were also anonymized after transcription. Participants did not review transcripts afterwards. Interviews were between 27 and 67 min long. Interview setting seemed to affect the length of the interview, as remote interviews were, in general, shorter. The semi-structured interview style allowed participants a degree of freedom in their account of the themes of discussion. However, clarifications asked by the interviewer may have influenced the direction of the interview.

The study employed qualitative content analysis (QCA). As a method, QCA offers a structured approach for analyzing large bodies of qualitative data [44]. Here, the interview data could be examined with a limited scope in mind. This scope is referred to as the “coding frame”. The coding frame was built around the research questions of this study, allowing for the identification of the attributes of sustainable packaging, the motivations behind packaging innovations, and the actors sharing the responsibility to steer innovation. These three are referred to as main categories, and they function as a tool for identifying and specifying relevant aspects, subcategories, from the material. The aim was to pay particular attention to variation within the data, i.e., to seek similarities and differences in the material. The analysis was data-driven, which in QCA often means that patterns were identified and categorized from the data [45]. The coding frame, namely the categories of attributes associated with sustainable food packaging, motivations behind innovation, and division of responsibilities in the innovation ecosystem, was based on the research questions of this study.

QCA has an embedded system of assessing internal reliability. This is specifically aimed at examining the reliability of the coding frame. The assessment is done via a comparison, either across different people or two at two different points in time. As the comparison for this study was done across two different points in time, the aspect of the coding frame that was assessed was its stability. The instrument used to assess reliability here was done through calculating a percentage of agreement across two points in time [44].

Here, the percentage of agreement was 71 percent, and was calculated by the difference between the first and second round of coding. Variation in the percentage of agreement could be caused by many factors, such as limitations of the coding frame, or human error. The complexity and richness of the interview data may also be a factor, as it could make the coding process more challenging. The meaning of material such as interview transcripts is rarely explicit, and a degree of uncertainty will always remain.

## Results and Discussion

This section of the article will present and discuss the findings of the study. Using qualitative content analysis, the types of attributes participants mentioned in the context of sustainability of food packaging were examined. Moreover, the motivation and responsibilities of actors were studied.

### Attributes Associated with the Sustainability of Food Packaging

As can be seen from Table 2, altogether 11 attributes associated with sustainability of packaging were identified. Mitigation of food loss and waste (1.1) emerged from the material more often than other attributes with 17 mentions across the transcripts. The emphasis placed on food loss and waste by the participants parallels findings made elsewhere, as the environmental impact of food packaging is dependent upon its impact on decreasing or increasing food losses (e.g., [5–7]). Participant number 3, a public servant, described the role of packaging in the following manner: “…the packaging, after all, is a way to reduce [food] waste” (3).

**Table 2:** Coding frequencies for subcategories of “attributes associated with sustainability of food packaging”

1. Sustainability of food packaging	1	2	3	4	5	6	7	8	9	10	11	12	13	14	Total
1.1. Mitigates food waste and loss	0	1	3	0	0	3	3	1	1	1	2	1	1	0	17
1.2. Full environmental impacts considered	0	0	2	0	1	3	1	2	0	0	5	1	0	0	15
1.3. Recyclability	0	0	1	0	0	0	5	1	0	0	1	0	0	5	13
1.4. Reduces plastic waste	0	0	0	0	3	1	0	0	0	0	0	1	2	0	7
1.5. Sustainable materials	3	0	0	0	0	0	0	0	0	2	0	1	0	0	6
1.6. Carbon-neutrality	0	0	0	0	0	0	0	4	0	1	0	0	0	0	5
1.7. Biodegradability	0	0	0	0	1	1	0	0	0	1	0	0	2	0	5
1.8. Improved efficiency	0	0	0	0	0	0	0	0	1	2	1	0	0	1	5
1.9. Sustainable product	0	1	0	0	0	0	0	2	0	1	0	0	0	0	4
1.10. Functionality	0	0	0	0	0	0	0	0	0	2	0	1	1	0	4
1.11. Novel materials	0	0	0	0	1	0	0	0	0	0	0	0	0	0	1

Half of the interviewees discussed the full environmental impact of food packaging (1.2), arguing that environmental impacts of packaging are complex (see Table 2, subcategory 1.2). An interviewee, working in an industry association, voiced the tensions between material improvements and food waste: “…we would need to be able to evaluate whether or not the food waste is less important than the improved recyclability of packaging. And from there, the assessment is not that simple anymore” (2).

The complexity of balancing different environmental impacts has been noted, as new methods of life-cycle analysis seek to integrate the entire packaging life cycle and the product-packaging relationship into assessments of sustainability (see, e.g., [32]). Similarly, Russell [38] points out that even more resource-intensive packaging alternatives, such as intelligent packaging, may be justified if their overall impact on the food supply chain is positive. It appears that the participants of this study hold a relatively holistic view of sustainable food packaging. Therefore, the perceptions of consumers and expert actors seem to conflict, as consumers are mainly concerned with the materials of food packaging [29].

Nevertheless, several participants (see categories 1.3 to 1.7, and category 1.11) mentioned material choices in food packaging. As can be observed from Table 2, out of these categories, recyclability (1.3) was mentioned most frequently. Recyclability, as argued by a senior development manager, was a crucial aspect of their research and development work: “[packaging innovation] is heavily focused on developing materials, on how we can make them recyclable, how we can add renewable and recycled materials” (8).

It is worth noting that several features of sustainable material choices appear in the results (see Table 2), with goals ranging from plastic reduction (1.4) to biodegradability of materials (1.7). This potentially suggests that internal disagreements over material solutions exist in the field. What is more, the division of responses also indicates the range of needs that exist within the food and beverage industries, as different combinations of materials and technologies are used to achieve different protective qualities [4].

While many different material attributes emerge from the interview data, they parallel observations made elsewhere: for example, Zhao et al. [14] discuss the trajectory of bioplastics use, driven by consumer interest in plastic waste, the availability of nonrenewable materials (petroleum), and improved technologies in production. Here, consumer demand and nonrenewable materials relate to the attributes identified in this study. Materials such as glass and metal are not mentioned by the interviewees, which was relatively surprising, as research indicates that consumers prefer these over, e.g., plastic for some food products [46]. This could also reflect the participants focus on food packaging production, where single-use materials are commonly used. Materials such as glass and metal are not mentioned by the interviewees, which was relatively surprising, as research indicates that consumers prefer these over, e.g., plastic for some food products [46]. This could also reflect the participants’ focus on food packaging production, where single-use materials are used more commonly. This could further suggest an existing juxtaposition between the views of consumers and the views of experts, but this could not yet be validated with the data used.

Some of these priorities seem to overlap to an extent, so the choice was made here to code data based on the how the problem, and its resolution are determined—for example, a public servant described the need for biodegradability of plastics in the following manner: “especially in developing countries, the mountains of trash caused by bottles are massive. So, beverage companies need to, for sure, develop new biodegradable products and to replace plastic with other solutions, or at least partly with other alternatives” (5). As biodegradability is presented as the solution to the problem, it was decided to be the determining attribute of sustainability in food packaging.

Four additional attributes were also observed in the interview data. These related to improving material efficiency across the value chain (1.8), the role of the product-packaging combination (1.9), and functionality in packaging use phase (1.10). The choice was made to handle categories 1.2 and 1.9 separately despite similarity of themes within them. Category 1.9 was coded in cases where the product itself was specifically mentioned by the interviewee.

The range of attributes associated with what constitutes sustainability of food packaging identified here does not reflect social and economic concerns related to sustainability in more general terms (as outlined by, e.g., Boons et al. [25]). Most often, the understanding of sustainability of packaging seems to focus on matters of food loss, material choices, and material recyclability. Attributes that could add understanding of social and economic sustainability have been outlined, albeit outside the context of sustainability-driven innovations. The social and economic dimensions of sustainability are by no means irrelevant. In the implementation of active packaging innovations, social sustainability was considered through raising consumer awareness, and improving product quality. Similarly, in the context of active packaging, economic sustainability was discussed in terms of improved efficiency in production [16]. Following this, it seems feasible that similar formulations could be made in the context of sustainability of food packaging.

### Motivations for Sustainable Innovation

The second dimension of this study was concerned with the motivations behind food packaging innovation. Often, when discussing innovations, the term “drivers” is preferred. However, the interviews here included questions on the personal significance of innovation for the interviewees. Therefore, the term motivation was chosen, to accommodate a variety of rationales behind innovation. Based on these findings, there seem to be two types of motivations: ones relating to the individual level, and others relating to business performance. At the individual level, motivations involve personal disposition towards innovation (2.1), interests in improving social aspects of sustainability (2.3), and concern over the environment (2.6). At the level of businesses, the identified motivations had to do with the existing business case for sustainable innovation (2.2), changing demand (2.4), regulatory compliance (2.5), added value from packaging (2.7), and changing industry norms (2.8) (Table 3).

**Table 3:** Coding frequencies for subcategories of “motivations for sustainable innovation”

2. Motivations for sustainable innovation	1	2	3	4	5	6	7	8	9	10	11	12	13	14	Total
2.1 Personal disposition	1	1	0	1	0	0	0	2	2	2	1	1	1	3	15
2.2 Business case for sustainability	3	0	0	2	1	0	0	4	1	2	1	0	0	0	14
2.3 Social sustainability	0	1	0	0	4	0	1	0	1	0	0	0	0	5	12
2.4 Changing demand	1	1	0	2	0	1	1	0	1	2	0	2	0	0	11
2.5 Compliance with regulation	0	2	1	0	0	1	0	1	1	1	1	1	0	0	9
2.6 Concern over the environment	0	0	0	4	0	1	0	0	0	0	1	1	1	1	9
2.7 Added value from packaging	0	0	0	0	0	3	0	0	3	0	0	0	0	0	6
2.8 New industry norms	1	0	0	0	0	0	0	0	1	1	0	0	1	0	4

It was possible to find factors that explain innovations through personal dispositions—interviewees here found innovation exciting, as is exemplified by a CEO working with packaging production, who argued it was rewarding to “combine things and create something new” (9).

Moreover, changes involving the society at large were also found in the data, with concerns over creating job opportunities, ensuring equal access to food, and ensuring a just transition away from resource-intensive industries. An interviewee who works in an industry association argued for social dimensions of innovation in this manner: “…the change that is coming with climate change is going to be painful – but it can’t hurt the most vulnerable people” (14).

Similarly, interviewees discussed having personal concerns over environmental degradation. For example, an interviewee working with plastic alternatives discussed their motivations to work with food packaging innovation in particular: “So, food packaging, it is at risk of ending up, or at a larger risk than many other things to end up in nature. And then, if you want to have a meaningful impact in the short term then it’s good to be there” (4). Literature on innovation has paralleled the sentiments expressed in the previous quotes—for example, Crossan and Apaydin [47] argue that the purpose of innovation is undergoing changes, as factors that go beyond profits or value-added novelty impact the decision-making processes of organizations.

Although social aspects of sustainability did not appear in the attributes assigned to sustainable food packaging, they appear in the motivations how actors indicate to direct their work. This could potentially simply be a feature of the interview guide, if questions have directed the responses to a particular way. For example, it could have changed the results if the three dimensions of sustainability had been addressed by the interviewer directly. Now, the responses were somewhat unprompted or spontaneous, but also perhaps more genuine, and in this way, the three dimensions—environmental, social, and economic—of sustainability do not emerge. However, it is interesting that although the three-dimensional understanding of sustainability is present in the motivations of actors in the sector, it does not emerge from packaging attributes alone.

Based on this interview data, it seems that the food packaging industry sees a business case for promoting sustainable food packaging innovation. For example, the interviewee quoted above on the environmentally conscious mindset on innovation also argued that food packaging is a lucrative market: “in the food packaging side, they use more than a half of all flexible packaging materials, so it is an attractive market for us” (4).

Product level sustainability simply made sense from the point of view of business, as described by a brand owner: “in some cases, we can combine [sustainability and business operations], for example making our materials thinner, it is good for both procurement and sustainability” (8). A CEO mentioned that the importance of sustainability has grown over time: “its significance is growing and companies have realized that it makes sense, really, it is beneficial for the business to do things sustainably” (9).

This trend might act as a catalyst for the packaging industry’s R&D efforts. The findings suggest that actors in the food packaging innovation ecosystem recognize the opportunities that are related to innovating for sustainable food packaging—a finding that is echoed by Keränen et al. [23]. The authors emphasize the importance of opportunity recognition, as it enables the wider diffusion of sustainable innovations.

In addition to the above, changes in demand structure for food packaging were found in analysis. Interviewee 1, who worked as a CEO in material development, argued that there have been vast changes in the trajectories of R&D in the past few years, through changes in demand. “Back then the talk was about ease-of-use (–). In the past few years this has turned into how well [food packaging] can be recycled, and how ecological packaging is” (1).

Similarly, interviewee number 4., also working with material development, discussed changes in demand: “…in 2015 we went to show our awful samples to our clients, they really were awful that time, have nothing to do with the fine product we have today, we already got the pull from there then. These clients [said] that this is what we need” (4). These statements echo some findings made in literature: for example, Zhao et al. (2020) identified a change in demand for plastic alternatives.

Moreover, one participant, working in the field of waste management, brought up the huge publicity the issue of marine plastic pollution has received. This, according to them, has had significant impact on food packaging and the surrounding industries: “the publicity plastic has received, publicity, oceans, plastics, and everything else, has woken consumers up and that way woken up those who sell packaged products and packaging producers. So, there we have seen a dimension that is important to buyers” (7). As argued by Vernuccio et al. [37], consumer behavior and the rise of new environmental values act as key drivers of food packaging innovations. These both are also present here, as consumers and the industry adopt new values regarding the environment and consumers readjust their preferences in packaging.

Compliance with (and anticipation of) regulation (2.5) emerged as a relatively important source of motivation for the actors in this study with nine mentions by eight interviewees. A CEO, working in material production, said their products are more attractive to potential buyers as they “provide economic benefits” (10) by fitting in with the current regulatory framework.

Therefore, the R&D processes are geared towards benefiting from existing regulation concerning packaging materials. EPR as a motivation was also mentioned by an industry association representative, who argued that EPR has morphed into a “price tag of responsibility” (2) as companies have sought out new solutions to optimize costs under existing regulations.

### Responsibility of Steering Innovation

As its third dimension, the study sought to understand how actors divide the responsibilities of steering innovation within the food packaging value chain. As can be seen from Table 4, six stakeholders were identified from the material: the Finnish government, brand owners in the food and beverage industry, private sector (voluntary action), consumers, private sector (compliance), and overseas governments. Table 4 also provides the breakdowns of answers, and categories are organized from the most to least often mentioned. The way actors share responsibility over steering innovation is interesting, as innovation ecosystems are highly connected—decisions made by a single actor can affect the entire network [48].

**Table 4:** Coding frequencies for subcategories of “responsibility to steer innovation”

3. Responsibility to steer innovation	1	2	3	4	5	6	7	8	9	10	11	12	13	14	Total
3.1. The Finnish Government	0	1	3	2	2	3	1	5	6	3	4	0	2	3	35
3.2. Brand owners	0	6	0	4	3	0	0	0	0	3	0	0	0	2	18
3.3. Private sector (voluntary action)	1	2	1	0	0	0	1	1	1	0	3	1	0	2	13
3.4. Consumers	0	0	0	0	0	0	0	3	0	2	0	0	1	0	6
3.5. Private sector (pre-emptive action)	0	1	0	0	0	1	0	0	0	0	0	0	0	0	2
3.6. Overseas governments	0	0	0	0	2	0	0	0	0	0	0	0	0	0	2

The role of the Finnish government is emphasized in the data collected for this study. One interviewee, a brand owner representative, noted that the electoral cycle is a big determinant in packaging regulation, particularly in terms of increasing or decreasing consumer duties: “politicians will not want to blame consumers (–) because then they will not be re-elected” (8). In this quote, it is possible to observe a wish to place more responsibility on consumers—however, this is seen as first and foremost a political challenge, or a duty of the government.

Similarly, the elected government was seen as an arbitrator of firm competitiveness, as described by a public servant working with innovation: “in this world of environmental challenges, I hope that [politicians] do not make it too difficult for enterprises” (5). Another public servant working with regulation concerning food packaging discussed their role in the system and argued that it is important to take down regulatory obstacles when materials are known to be safe: “we can still advance legislation [in the European Union] to make [new innovations] possible” (3). Governmental organizations do play a role—for example, the market introduction of active packaging solutions is hindered by the slow approval processes that are put in place to ensure food safety [16].

Brand owners in the food and beverage industries were also deemed responsible by the interviewees with altogether 18 coded instances. The responsibility was perceived to lie within their ability to influence the demand for new food packaging alternatives. An interviewee, working with new packaging materials, described brand owner responsibility and risk-taking in the following manner: “[Companies working with new food packaging alternatives] can’t [wait] until the product has passed all the tests and production has been scaled. So it has its risks, and that’s why what we should do, is to inject some courage into food and beverage companies, the brand owners” (4).

This risk-taking can be seen to fall as a duty of brand owners to make decisions that impact the demand for new materials and new types of packaging, with the value chain before them being rather powerless in the equation. In their study, Keränen et al. [23] also note that brand owners can make innovation more challenging—suppliers of packaging often need to be able to offer the same solutions to all their customers to ensure equal treatment. Therefore, innovations to respond to specific needs may be neglected in order to ensure continued relationships with customers. However, Keränen et al. [26] also report that brand owners perceive material suppliers to challenge the wider use of bioplastics in food packaging.

It is possible that the roles of brand owners and the government intersect to an extent. For example, Olsmats and Kaivo-oja [49] argue that the packaging industry (i.e., those in the value chain before brand owners) acts reactively to new changes imposed by the government, or new demands from brand owners. Here, brand owners emerged as particularly important in terms of their ability to influence the demand for certain types of packaging.

Consumers and their role in the division of responsibility over value-chain actors were also apparent from the interview material. Consumers in this complex exist as a sort of unpredictable entity, with certain preferences for packaging that end up being outpriced by less innovative packaging. An interviewee, a senior development manager of a brand owner, described consumer behavior in the following manner: “…everyone in surveys say they [are willing to pay] but in reality, when you are standing [in a grocery store] then you just look at the price per kilogram” (8) indicating a perception of the contradictory preferences of consumers, and their impact on packaging development. Similar contradictions have been noted by others: for example, Werner et al. [17] assert that consumers simultaneously possess negative opinions on the environmental impact of packaging, and demand convenient, healthy, and safe food products. This is a challenge for the industry, as successful packaging innovations require consumer acceptance. Furthermore, Tiekstra et al. [16] note that consumer acceptance is key in introducing new packaging alternatives.

In terms of the role of private sector actors, interviewees mentioned the role of voluntary action and changes to operations. One interviewee from a packaging manufacturer suggested the adoption of new platforms, and “adopting new forms of systematic work” (12) in steering sustainable innovations in the food packaging industry. Another participant, an interviewee from a material producer mentioned that “[they] want to be a part in directing and changing the industry, the packaging industry, to a more sustainable direction where… (–) discussion becomes more rational” (1).

Here, willingness to move into more ecosystem-like ways of working could be beneficial, with firms benefiting from a range of capabilities and working towards a shared goal. As argued by Oksanen and Hautamäki [22], stakeholders of innovation processes may benefit from an environment where they can find their role. This could aid in defining the existing problems and necessary solutions to global challenges. While Keränen et al. [26] focus on the role of value networks, they too emphasize the importance of collaboration among value chain actors. Similarly, Lopes et al. [18] noted that stakeholder collaboration in the complex food and beverage packaging systems is important. The results of this study parallel these findings; as actors already share similar interests and goals, improved collaboration could aid by bringing together a range of capabilities and know-how. This study identified various issues actors are interested in, from the mitigation of food loss and waste to plastic pollution.

This study contributes to developing sustainable innovation processes in the packaging context. Action is needed in order to overcome not only environmental issues of packaging, such as resource use, plastic pollution, and the global levels of solid waste and to highlight the contribution of packaging in reducing food loss and waste and ensuring food safety, but also to include socioeconomic aspects of sustainability in the innovation processes. Thus, the innovation ecosystem needs to find ways to facilitate collaboration and learn to share the responsibility between different actors who may hold different views on sustainability of packaging. Finally, this study argues that integration of holistic sustainable innovation in the packaging sector is necessary, as problem-solving may be left shallow if nothing is done to address structural issues in the packaging sector and food and beverage industries.

## Conclusions

This study elucidated how sustainability of food packaging is perceived in terms of its attributes, based on the analysis of interviews conducted with ecosystem actors. The goal was to study different aspects of sustainability in food packaging innovation processes. To do this, the study examined what elements of sustainability were considered important, what motivated innovation activities in the food packaging sector, and how the actors divided responsibilities for steering the ecosystem. The study addressed three questions: *what types of attributes do experts associate with food packaging*, *what factors motivate food packaging innovation,* and *how is responsibility divided among actors in the innovation ecosystem and stakeholders outside it?* Together, these questions build new understanding on the priorities of the innovation ecosystem among stakeholders and elucidate the importance of the ecosystem governance towards sustainability.

The most important *attributes* associated with food packaging revolve around preventing food waste and loss and considering environmental impacts as a whole. This implies that packaging business cannot be developed in a vacuum but as a part of the entire food system, where sustainable packaging has an instrumental role for sustainability. The most important *motivations* were outlined in terms of personal disposition and the business case for sustainability. This is somewhat conflicting with the views on who is *responsible for steering innovation*, where the Finnish Government was perceived as the most important actor. It is worth asking if the government can be expected to steer innovation if primary motivations are personal or have to do with sustainability being good for business.

The findings of this empirical study suggest that several things affect sustainable innovation in the food packaging sector. Firstly, actors in the ecosystem are able to recognize many aspects of ecological sustainability in packaging. They are, however, less concerned with factors beyond ecological sustainability. Different dimensions of sustainability—economic and social sustainability—do not emerge in relation to perceived sustainability attributes of food packaging. However, other facets of sustainability underscore the motivations actors have in working on packaging innovations. Secondly, motivations to develop new and more sustainable food packaging innovations include reasons from both the level of the individuals working with packaging and the level of the enterprise. Personal motivation, aspirations to improve social sustainability, and concerns over the environment are identified among the interviewees. From a business development standpoint, interviewees identify motivations that range from good business cases for sustainability to influencing market demand. Finally, overwhelming emphasis is found to be placed on government action to take responsibility for steering the innovation ecosystem towards sustainable innovation.

As a limitation, this study examined the phenomenon of sustainable innovation in a very specific context, namely in Finnish food packaging industry. The study also comes at a time when many processes are ongoing within the EU—for example, the single-use plastic ban became effective in 2021, which is likely to affect the data collected here. This is however not uncommon in the case of qualitative research [39]. Although the data represents a specific moment in time, changes in the industry are not rapid. Furthermore, it is worth emphasizing that the generalizability of the qualitative case-specific results may be limited, and further research is still needed.

This study supports the view that increased collaboration activity could improve the emergence of new sustainability-driven innovations in the food packaging sector. Actors seem to share goals—as they appear acutely aware of the importance of preventing food loss and waste. However, the roles and responsibilities do not appear to be clearly defined, as the government is seen primarily responsible for encouraging sustainable packaging innovation. These results have implications for policy-makers—clearly, a well-established platform for the ecosystem actors is needed to discuss and clarify the roles within the value-chain. Moreover, the findings suggest some attributes the expert interviewees associate with sustainable food packaging divert from those identified by consumers. Firstly, the participants of this study are heavily concerned with the mitigation of food waste and loss. As discussed above and in the literature, consumers are unaware of the role food packaging has in reducing food waste. This requires the practitioners of the industry to better understand and communicate the sustainability of food packaging with consumers. This has important managerial implications: reducing food waste through packaging innovation could provide a crucial avenue for mitigating the cumulative environmental impacts of the food system. Making choices based on material efficiency, circularity, and renewability could also have an impact on the global environment, while substituting fossil-based materials. This provides an opportunity for the packaging industry to incorporate sustainability into the value chain.

A more comprehensive approach to the packaging sector could be beneficial in future research. For example, studies on the retail sector could aid in understanding current brand-owner attitudes and consumer preferences. Moreover, better understanding of logistical requirements of especially fresh food packaging could explain some requirements placed on food packaging innovation and improve understanding of necessary technical properties of materials. Finally, studies that examine the gap between consumer and expert perceptions on food packaging could help in encouraging sustainable consumption and better consumer knowledge on the purpose of food packaging. Future research should also focus on understanding the complex dynamics of the packaging ecosystem, as the industry is built around both small, agile firms and large multinational corporations and their interactions.

More attention on packaging innovation is needed for sustainability-driven innovations to mainstream in the markets. As this article has outlined, the sector is undergoing changes from several directions, and the way these changes are responded to can contribute to improving the sustainability of the food system through food loss and waste prevention, encouraging recycling, and reducing the use and extraction of raw materials. This study offers an avenue for understanding what attributes of sustainable packaging are prioritized, and how innovation ecosystem actors and stakeholders perceive sustainability in the ecosystem itself. Whether the challenge is aging consumers, raw material availability, or changing consumer preferences, discussions across the ecosystem and its stakeholders are needed.

## Appendix

1. Interview guide

*Background*

1. What is your field of work? How would you describe your role in your organization?
2. How long have you worked in your field?
3. How would you describe your background?

*Innovation*

4. How would you describe the food packaging related innovation activities you encounter in your work?
  -  Do you think it is more related to single product innovations or to system-wide change?
5. How would you describe the innovation ecosystem you work in?
  -  How many value chain actors, on average, do you work with when developing an innovation?
  -  Are there more actors forward or backward in the value chain?

*Risks associated with innovation*

6. Could you describe the risks you associate with food packaging innovation currently?
  -  What kind of risks do you see in the future?

*Benefits associated with innovation*

7. Could you describe the benefits you associate with food packaging innovation currently?
  -  What kind of benefits do you see in the future (e.g. within the next 10 or 30 years)?

*Purpose of innovation*

8. What are the most central reasons for developing food packaging related innovation activities in your work?
9. Would you say the members of your organization share the same reasons?
  -  Which units have disagreements?
  -  Which units work well together?
  -  If disagreement: Do you think the disagreements are a resource or an obstacle for innovation?

*Significance of innovation*

10. What is the goal of innovation activity in your organization?
  -  What role does sustainability play?

11. What significance does innovation have in your life?

*Policy*

12. Which policy instruments limit food packaging innovation?
13. What kind of policies are needed to drive innovation in the field of food packaging?
14. How could innovation be made easier in your field through politics or policy-making?
  -  How could innovation be made more sustainable through politics or policy-making?
